# HIV-associated rectovaginal fistulae in children: a single-centre retrospective study in the antiretroviral era

**DOI:** 10.1007/s00383-024-05762-5

**Published:** 2024-07-08

**Authors:** Piero Alberti, Christopher Westgarth-Taylor, Emanuele Trovalusci, Robyn Charlton, Giulia Brisighelli

**Affiliations:** 1 Department of Paediatric Surgery, Chris Hani Baragwanath Academic Hospital, Faculty of Health Sciences, University of the Witwatersrand, Johannesburg, South Africa; 2 Paediatric Surgery Unit, Department of Women’s and Children’s Health, University of Padova, Padua, Italy; 3 Department of Paediatrics, Charlotte Maxeke Johannesburg Academic Hospital, Faculty of Health Sciences, University of the Witwatersrand, Johannesburg, South Africa

**Keywords:** HIV/AIDS, Rectovaginal fistula, Immunosuppression, Antiretroviral treatment, Pull-through

## Abstract

**Purpose:**

Acquired rectovaginal fistulae (RVF) are a complication of paediatric HIV infection. We report our experience with the surgical management of this condition.

**Methods:**

We retrospectively reviewed the records of paediatric patients with HIV-associated RVF managed at Chris Hani Baragwanath Academic Hospital (2011–2023). Information about HIV management, surgical history, and long-term outcomes was collected.

**Results:**

Ten patients with HIV-associated RVF were identified. Median age of presentation was 2 years (IQR: 1–3 years). Nine patients (9/10) underwent diverting colostomy, while one demised before the stoma was fashioned. Fistula repair was performed a median of 17 months (IQR: 7.5–55 months) after colostomy. An ischiorectal fat pad was interposed in 5/9 patients. Four (4/9) patients had fistula recurrence, 2/9 patients developed anal stenosis, and 3/9 perineal sepsis. Stoma reversal was performed a median of 16 months (IQR: 3–25 months) after repair. Seven patients (7/9) have good outcomes without soiling, while 2/9 have long-term stomas. Failure to maintain viral suppression after repair was significantly associated with fistula recurrence and complications (φ = 0.8, *p* < 0.05).

**Conclusion:**

While HIV-associated RVFs remain a challenging condition, successful surgical treatment is possible. Viral suppression is a necessary condition for good outcomes.

## Introduction

The HIV/AIDS pandemic has changed the face of medicine over the last 40 years. Not only has it caused significant morbidity and mortality, it has given rise to new diseases and increased the prevalence of previously rare clinical pathologies [1]. Diseases of the anus and rectum are particularly common in HIV-infected individuals and anorectal pathology has been estimated to account for about 40% of surgical presentations in HIV-infected individuals [2, 3].

The association between HIV infection and development of acquired rectal fistulae has been recognised since the 1990s [4–9].While the pathogenesis of HIV-related rectovaginal fistulae (RVF) is unknown, they are tough to arise on the background of infections of the anal glands (crypt abscesses) caused by HIV-related mucosal inflammation and protozoal infestation. Crypt abscesses likely rupture following an acute insult such as diarrhoeal illness, forming a perianal abscess that later tracks anteriorly to form a fistula with the urethra in males or the vaginal wall in females [2, 5].

While the vast majority of rectal fistulae in children are congenital in origin, belonging to the spectrum of anorectal malformations (ARM), HIV infection is one of the possible causes of acquired fistulae together with trauma, inflammatory bowel disease (IBD), and complications following surgery for Hirschsprung’s disease (HD) [10]. HIV infection has been recognised in the literature as the most common cause of acquired rectovaginal fistula (RVF), to the extent that some authors have suggested that the finding of an acquired RVF in a child should prompt testing for HIV even in the absence of other HIV indicator conditions, especially when maternal HIV status is unknown [10, 11].

The burden of HIV/AIDS in South Africa is high, with 8.45 million infected citizens as of 2022; more than half of whom are women and around 270,000 of whom are children [12, 13]. As such, paediatric surgeons working in the South African context can expect to encounter HIV-associated RVFs in their practice. Prior to the introduction of antiretroviral therapy, surgical outcomes from management of HIV-associated RVF were disappointing due to poor wound healing and high rates of dehiscence secondary to deterioration in immune function. A meta-analysis of studies in children with acquired RVF observed a prohibitively poor fistula closure rate of 20% [10]. Consequently, most authors advocated a conservative approach aiming at managing comorbidities and perineal excoriation, with surgery reserved for patients requiring a diverting colostomy for obstruction or life-threatening perineal infection [5, 14, 15].

The value of this traditionally non-surgical approach to HIV-associated RVFs has been called into question, since the availability of highly active antiretroviral therapy (HAART) allows to achieve viral suppression in patients with optimal treatment adherence. It is estimated that 63% of South Africa’s HIV-infected children are currently receiving HAART [13]. Studies in South Africa have shown that outcomes from surgical treatment of anorectal pathology in children have improved significantly with the introduction of HAART and that elective correctional surgery can be performed safely and without an increased risk of complications [16, 17]. However, no study has yet looked specifically at how the introduction of HAART may impact the treatment strategies and outcomes’ from paediatric HIV-associated RVF. We describe our experience with managing HIV-positive children with acquired RVFs. This will contribute to expanding the currently limited body of available knowledge, which will ultimately be required to develop updated recommendations on the management of this condition.

## Materials and methods

After obtaining ethics approval (M2000616), a single-centre retrospective review of female paediatric patients (1 month to 13 years of age) with HIV-associated rectovaginal fistulae who were seen and managed by the Paediatric Surgery Department at Chris Hani Baragwanath Academic Hospital (CHBAH) between 1 January 2011 and 31 December 2023 was performed.

Participants for the study were selected among patients admitted as inpatients to the CHBAH paediatric surgery ward and patients attending the CHBAH paediatric colorectal outpatient department (OPD). Female patients aged 1 month to 13 years with human immunodeficiency virus (HIV) infection confirmed by polymerase chain reaction (PCR) assay (if below 18 months of age), or enzyme linked immunosorbent assay (ELISA) (if above 18 months of age) who developed RVF after birth were included in the study. We excluded patients with RVF observed at birth or prior to development of HIV infection, patients with acquired RVF secondary to other causes (e.g. trauma, IBD, post pull-through for Hirschsprung’s disease), and patients with substantially incomplete records (e.g., no surgical history available).

Demographic data, contact details, and information about patients’ surgical histories, HIV viral loads and CD4 counts were collected from patients’ digital health records and by retrieving hard copies of their admission records from the CHBAH archives. Patients were contacted telephonically, and information was collected about their current continence status, bowel habit, and soiling episodes. Contacted patients were invited to attend in-person follow-up at the local colorectal clinic. Outpatient files were consulted for patients who attended in-person follow-up during the study period.

A ‘surgical procedure’ was defined as any intervention for which a patient required a general anaesthetic. Patients’ HIV viral loads were defined as ‘undetectable’ if < 100 copies/ml, suppressed if < 1000 copies/ml, and unsuppressed if > 1000 copies/ml, in accordance with WHO guidance [18]. Absolute CD4 counts in cells/μl were used to classify patients as having stage 1 (low risk), 2 (moderate), or 3 (AIDS) disease based on 2021 WHO guidelines on infant diagnosis of HIV (see Appendix 1) [19–21].

Data were entered into a chart created for this study in Microsoft Excel (Microsoft, Santa Rosa, California, USA, 2021). Statistical analyses were performed using GraphPad 9.0.0 (GraphPad Software, San Diego, California, USA, 2024). Mean and standard deviation were used to present normally distributed data. Median and interquartile range (IQR) were used if data did not follow a normal distribution. Two-tailed Student’s t test was used for analysis of continuous data. Two-tailed Fisher’s exact test was used for analysis of categorical data. Statistical significance was defined as *p* < 0.05.

## Results

**Fig. 1:**
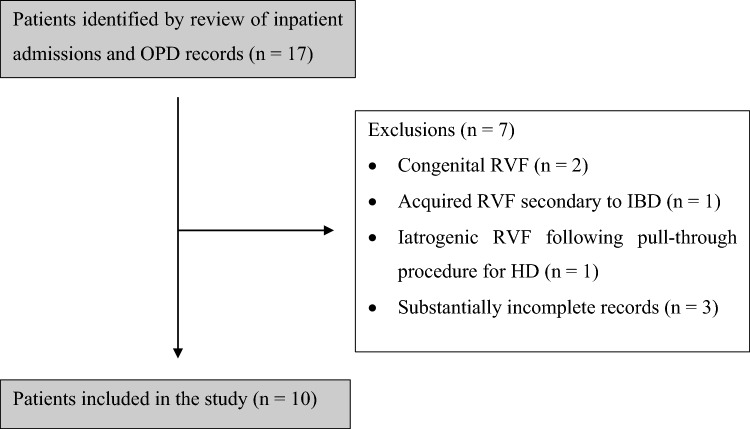
Patient selection flowchart

An initial review of inpatient admission records and records from the colorectal OPD identified 17 patients with rectovaginal fistulae as potential participants to the study. Seven patients were subsequently removed based on exclusion criteria, leading to study population of ten patients (*n* = 10) (Fig. 1). Treatment trajectories for included patients are summarised in Table 1.

**Table 1:** Overview of treatment trajectories for HIV-associated RVF (patients ordered by date of birth)

Patient	Presentation	Fistula repair	HIV control after repair	Complications	Outcome	Surgeries
1	Vaginal passage of stool Age: 7 years 4 months HIV: undetectable viral load, CD4 889 cells/μl	Posterior sagittal anorectoplasty Age: 7 years 7 months HIV: undetectable viral load, CD4 900 cells/μl	Maintained	None	Stoma closed at 7 years 10 months Currently 15 years 7 months Continent, not soiling	**3** (0 EUA)
2	Perineal sepsis Age: 2 years 1 month HIV: undetectable viral load, CD4 524 cells/μl	Posterior sagittal anorectoplasty Age: 2 years 6 months HIV: undetectable viral load, CD4 1579 cells/μl	Maintained	None	Stoma closed at 3 years 6 months Currently 14 years 8 months On daily rectal washouts, not soiling	**4** (1 EUA)
3	Vaginal passage of stool Age: 1 year 1 month HIV: viral load 185 copies/ml, CD4 1,837 cells/μl	Anterior rectal wall pull-through Ischiorectal fat pad interposed Age: 6 years 6 months HIV: undetectable viral load, CD4 1899 cells/μl	Failed VL 13,095 CD4 443	One recurrence Anal stenosis Wound sepsis Perineal sepsis	Currently 13 years 7 months Permanent stoma due to damaged sphincters and dentate line	**24** (15 EUA)
4	Perineal sepsis Age: 2 years 11 months HIV: viral load 243,330 copies/ml, CD4 733 cells/μl	Anterior rectal wall pull-through Ischiorectal fat pad interposed Age: 3 years 6 months HIV: viral load 406 copies/ml CD4 1395 cells/μl	Failed VL 4500 CD4 1010	Two recurrences Perineal sepsis	Stoma closed at 5 years 7 months Currently 11 years 5 months Continent, not soiling	**13** (4 EUA)
5	Vaginal passage of stool Age: 3 years 0 months HIV: viral load 787,441 copies/ml CD4 1,159 cells/μl	Anterior rectal wall pull-through Ischiorectal fat pad interposed Age: 3 years 11 months HIV: viral load 109 copies/ml 1750 cells/μl	Failed VL 7244 CD4 872	Two recurrences Anal stenosis	Currently 11 years 3 months Permanent stoma, lost to follow-up before reversal	**8** (3 EUA)
6	Vaginal passage of stool Age: 2 years 1 month HIV: undetectable viral load, CD4 454 cells/μl	Anterior rectal wall pull-through No fat pad interposed Age: 6 years 2 months HIV: undetectable viral load, CD4 1056 cells/μl	Maintained	One recurrence	Stoma closed at 9 years 0 months Currently 11 years 2 months Continent, not soiling	**12** (3 EUA)
7	Vaginal passage of stool Age: 6 months HIV: viral load > 10 million copies/ml, CD4 232 cells/μl	N/A	N/A	N/A	Demised at 10 months of age	**0**
8	Vaginal passage of stool Age: 2 years 4 months HIV: viral load 4,787,230 copies/ml CD4 214 cells/μl	Anterior rectal wall pull-through Ischiorectal fat pad interposed Age: 4 years 0 months HIV: undetectable viral load, CD4 2166 cells/μl	Maintained	None	Stoma closed at 5 years 8 months Currently 8 years 3 months Continent, not soiling	**7** (3 EUA)
9	Perineal sepsis Age: 1 year 9 months HIV: viral load 19,622 copies/ml, CD4 292 cells/μl	Anterior rectal wall pull-through and perineal reconstruction No fat pad interposed Age: 4 years 0 months HIV: viral load 299 copies/ml, CD4 1266 cells/μl	Failed VL 9309 CD4 933	Perineal sepsis	Stoma closed at 5 years 4 months Currently 6 years 10 months Continent, not soiling On long-term topical treatment for perineal excoriation	**5** (1 EUA)
10	Vaginal passage of stool Age: 9 months HIV: 1,667,675 copies/ml, CD4 432 cells/μl	Anterior rectal wall pull-through Ischiorectal fat pad interposed Age: 5 years 10 months HIV: viral load 135 copies/ml, CD4 817 cells/μl	Maintained	None	Stoma closed at 5 years 11 months Currently 6 years 8 months Continent, not soiling	**6** (3 EUA)

Bold vlaues indicate the total number of surgeries required by the included patients

### Patient characteristics at presentation

All patients in the study had confirmed HIV infection. Six patients (6/10) were diagnosed by PCR and four (4/10) by ELISA. Patients were diagnosed with HIV at a median age of 10 months (IQR: 1 month to 2 years 3 months). All patients were initiated on HAART in accordance with South African guidelines on management of HIV in paediatric patients [22].

Acquired RVF was a presenting feature of HIV disease for four (4/10) patients—one patient developed an RVF in their first year of life but was only formally diagnosed with HIV at the age of 2 years. The remaining 6/10 patients presented with an acquired RVF a median of 14.5 months (IQR: 10–23 months) following their HIV diagnosis.

The median age of presentation with RVF across all included patients was 2 years (IQR: 1–3 years). The youngest and oldest ages of presentation were 6 months and 7 years 4 months, respectively. Seven (7/10) patients presented with vaginal passage of stool and three (3/10) with perineal sepsis. Eight patients (8/10) were managed at CHBAH throughout their treatment period, while two patients underwent part of their treatment at other institutions.

Patients’ median HIV viral load at presentation with RVF was 19,622 copies/ml (IQR: undetectable to 1,227,558 copies/ml). Six patients (6/10) presented with an unsuppressed viral load. Viral load at presentation was suppressed (< 1000 copies/ml) in one patient (1/10) and undetectable (< 100 copies/l) in 3/10 patients. Patients’ median CD4 count at presentation was 524 cells/μl (IQR: 362 cells/μl to 1024 cells/μl). Based on their ages, CD4 counts, and comorbidities at presentation, one patient (1/10) was classified as having stage 1 HIV disease (low risk), while 9/10 patients were classified as having stage 3 disease (AIDS). Of note, the three patients who presented with an undetectable viral load were still classified as having stage 3 disease due to previous history of AIDS-defining illnesses, which included HIV encephalopathy, extrapulmonary tuberculosis, and CMV colitis (see Appendix 1).

### Surgical management

Nine patients (9/10) underwent surgical management of their RVF. One patient presented at the age of 6 months with multiple issues including fungal sepsis, encephalopathy, and vaginal passage of stool. Colostomy was not attempted in the index admission as the patient was judged too unwell to undergo surgery. The patient improved and was discharged but later developed chronic pulmonary disease requiring readmission. She demised from bronchopneumonia at the age of 10 months before any surgical management could be initiated.

A total of 82 procedures were performed for the patients who underwent surgical management. The median number of required surgeries per patient was of 7 procedures (IQR: 4.5–12.5 procedures) (Table 2). All patients underwent a diverting colostomy prior to RVF repair. None of the patients showed spontaneous RVF resolution following faecal diversion. Seven patients (7/9) had their stoma permanently reversed, while two patients (2/9) have long-term stomas.

**Table 2:** Surgical procedures performed for patients with HIV-associated RVF, according to recurrence

Procedure	Surgical trajectory		All patients (*n* = 9)
	RVF recurrence (*n* = 4)	No recurrences (*n* = 5)	
Stoma fashioning	4	5	9
Re-do stoma fashioning	4	0	4
Stoma revision	5	1	6
Biopsy of rectum or stoma site	3	1	4
Anterior rectal wall pull-through	4	3	7
Posterior sagittal anorectoplasty	0	2	2
Re-do pull-through	3	0	4
Perineal body reconstruction	3	0	2
Stoma reversal	4	5	9
Emergency laparotomy for wound sepsis or adhesive bowel obstruction	2	0	2
Examination under anaesthesia (EUA)	25	8	33
Total	**57** (Median: 12.5, IQR: 10 to 18.5)	**25** (Median: 5, IQR: 6.5 to 3.5)	**82** (Median: 7, IQR: 4.5 to 12.5)

Bold vlaues indicate the total number of surgeries required by the included patients

Fistula repair was performed a median of 17 months (IQR: 7.5–55 months) following colostomy, whereas definitive stoma reversal was performed a median of 16 months (IQR: 3–25 months) after initial fistula repair. Patients whose RVF recurred after the initial repair required 9.3 additional procedures (95% CI 1.9–16.6 procedures, *p* = 0.02) and had their stoma reversed 19.5 months later (95% CI 2.6–36.4 months, *p* = 0.03) compared to patients who did not experience recurrences. No significant difference in number of surgeries required or time from fistula repair to stoma reversal was observed based on patients’ history of complications such as anal stenosis or sepsis.

**Fig. 2:**
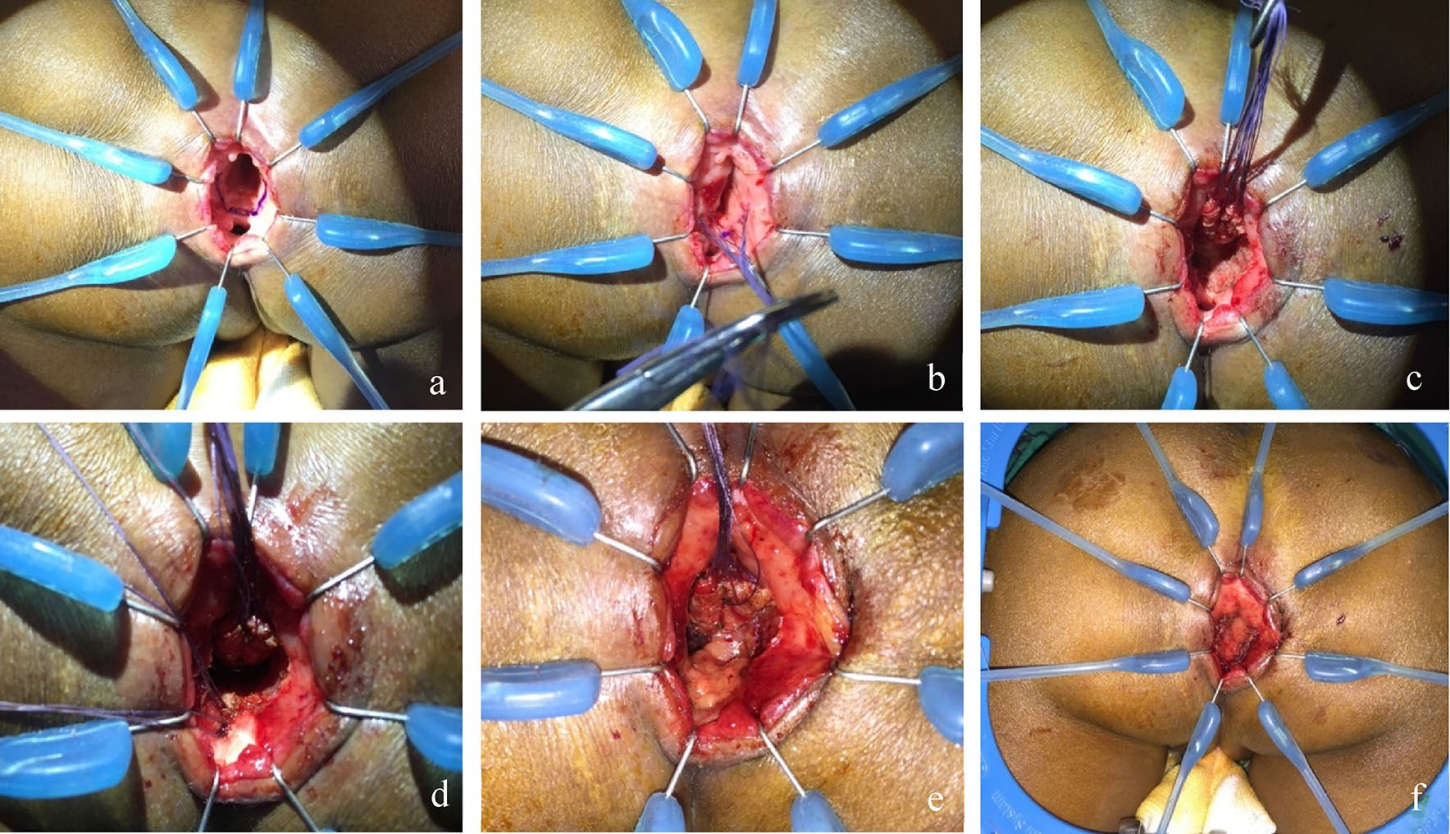
Steps to transanal RVF repair. **a** Insertion of Lone Star^®^ retractor and fistula visualisation. **b** Placement of stay sutures on the anterior rectal wall. **c** Mobilisation of the anterior rectal wall. **d** Closure of the fistula tract. **e** Ischiorectal fat pad interposition. **f** Advancement flap pull-through and anastomosis

A posterior sagittal anorectoplasty was performed to repair the RVF for the first two patients in the study. Fistula repair in all subsequent patients was performed by a transanal anterior rectal wall pull-through, leaving the perineal body intact. After inserting a Lone Star^®^ retractor at the mucocutaneous junction, the fistula is identified and the mucosal lining is debrided. Stay sutures are placed on the anterior wall of the rectum proximally to the fistula. Using needle-tip diathermy, an incision is made distal to the stay sutures and deepened. The fistula is closed with interrupted absorbable sutures. The anterior rectal wall is then pulled through using an endorectal advancement flap to cover the repair [23]. An ischiorectal fat pad can be interposed between the fistula repair and the anterior rectal wall before the anastomosis is performed (Fig. 2). An ischiorectal fat pad was interposed in the initial fistula repair for five (5/9) of our patients. Fat pad interposition in the initial repair was not observed to have a significant association with risk of subsequent recurrence. A fat pad was always interposed in procedures for the repair of recurrent fistulae [24].

Patients only underwent fistula repair once their viral load had been suppressed (Table 3). Patients’ median viral load at the point of fistula repair was undetectable (IQR: undetectable to 217 cells/ml) and their median CD4 count was 1395 cells/μl (IQR: 978 cells/μl to 1824.5 cells/μl). No significant difference in viral loads and CD4 counts at presentation and at fistula repair was observed based on patients’ later history of recurrence or complications.

**Table 3:** HIV treatment trajectories in patients with surgically managed RVF

	Median viral load (copies/ml) [IQR]	Median CD4 count (cells/μl) [IQR]
Presentation (*n* = 9)	19,622 [undetectable to 1,227,558]	524 [362–1024]
Stoma fashioning (*n* = 9)	389 [undetectable to 1,227,558]	733 [443–1024]
RVF repair (*n* = 9)	Undetectable [undetectable to 217]	1395 [978–1824.5]
Repair of first recurrence (*n* = 4)	Undetectable in all patients	1566 [1045–1989]
Repair of second recurrence (*n* = 2)	Undetectable in both patients	1693–2312
Stoma reversal (*n* = 7)	Undetectable [undetectable to 135]	1349 [817–1914]

### Complications and outcomes

The most common complications in our patients were recurrence of the fistula, anal stenosis, and wound or perineal sepsis. Four patients (4/9) had RVF recurrences, which were observed a median of 5 months (IQR: 4–6 months) after the preceding repair. Two patients had a single recurrence and two patients had two recurrences, for a total of six recurrences. Three recurrent fistulae were repaired by re-do pull-though with ischiorectal fat pad interposition. In the other three cases, recurrent fistulae were associated with perineal disruption and damage to the anal sphincters. Fistula repair was therefore combined with perineal body reconstruction through a posterior sagittal approach. Overall, 15 procedures for RVF repair were performed.

Two patients (2/9) developed stenosis of the anal canal as a complication of fistula repair. Anal stenosis was satisfactorily managed with serial anal dilatations in one of the patients, while the other patient developed a long-standing anal stricture and was subsequently lost to follow-up.

Sepsis was observed in 3/9 patients. Two patients developed perineal sepsis due to wound dehiscence following fistula repair, while one patient developed both perineal sepsis following fistula repair and laparotomy wound sepsis as a complication of stoma revision.

Patients were followed up for a median of 34 months (IQR: 14–84 months) after their last repair procedure. Three patients (3/9) attended in-person follow-up during the study period and three patients (3/9) were contacted telephonically. The remaining 3/9 patients could not be contacted and were classified as lost to follow-up. Information on their functional outcomes was obtained from the last available clinic note. Seven patients (7/9) have had good long-term outcomes, defined as stoma reversal and continence without any soiling episodes. Specifically, three patients (3/9) have remained continent since stoma reversal with no soiling, while four patients (4/9) experienced soiling episodes relating to constipation. These were treated with courses of bowel management with rectal washouts and laxatives: three patients have remained continent after discontinuation of bowel management, while one patient is clean on long-term daily rectal washouts. One patient is also on long-term topical treatments, having suffered extensive perineal excoriation due to sepsis.

Two patients (2/9) have long-term stomas and were classified as poor functional outcomes. The first patient had multiple complications, including recurrence, anal stenosis, and perineal sepsis. Colostomy reversal was performed with a covering ileostomy due to a short inflamed rectal stump. The patient was discharged, but represented the following month with symptoms of bowel obstruction which required an emergency laparotomy. Following multiple relooks, a colectomy and a long-term ileostomy were performed. The patient currently has extensive damage to the anal sphincters and disruption of the dentate line due to previous perineal sepsis and was thus judged unsuitable for stoma reversal. In the second patient, colostomy reversal was performed after a recurrent RVF was repaired by re-do pull-through. After 4 months, she represented with a second recurrence and a colostomy was refashioned. Re-do fistula repair and perineal body reconstruction were performed but were later complicated by development of a persistent anal stricture. This was being managed by serial anal dilatations, but the patient was subsequently lost to follow-up before her stoma could be reversed.

Patient’s surgical outcomes were not observed to significantly correlate to their HIV status at presentation. Patients who presented with an unsuppressed viral load did not differ from patients who presented with a suppressed viral load in overall number of surgical procedures, delays to fistula repair and stoma reversal, rate of recurrence or complications, and functional outcomes. By contrast, post-operative maintenance of viral control was significantly associated with surgical outcomes. Five patients (5/9) maintained a suppressed viral load following fistula repair, while four patients (4/9) were noted to have an unsuppressed viral load at some point after repair (Table 1). Failures in viral suppression occurred a median of 12.5 months (IQR: 7–16 months) after the previous repair. Compared to patients who maintained viral suppression after fistula repair, patients who experienced post-operative failures of viral suppression were noted to have a significantly higher rate of recurrence or complications including anal stenosis and sepsis (Table 4).

**Table 4:** Viral suppression and surgical outcomes (fistula recurrence, anal stenosis, or sepsis)

	Recurrence and/or complications	No recurrences or complications	Total
Not suppressed after repair	4	0	4
Suppressed after repair	1	4	5
Total	5	4	9

φ correlation coefficient = 0.8, *p* < 0.05

## Discussion

HIV-associated rectovaginal fistula is an important clinical entity for paediatric surgeons practicing in countries with a high prevalence of HIV infection in children. To our knowledge, this represents the largest case series in the post-antiretroviral treatment era. Over a period of 12 years (2011–2023), we identified ten patients who met all criteria for the study. By contrast, the largest study in the pre-antiretroviral paediatric literature, which was also based in a tertiary centre in South Africa, identified 37 patients over a period of 5 years (1996–2001) [15]. It is possible to speculate that wider availability of antiretroviral treatment may have made HIV-associated RVF a comparatively rarer pathology, one seen in children with advanced and untreated HIV disease. This hypothesis is supported by the finding that 9/10 of our patients were classified as having AIDS at presentation. Although 4/10 patients also presented with a suppressed viral load, it is possible that these patients developed RVF when their HIV was uncontrolled and that their viral control was then normalised before they presented to our centre. This is consistent with the observation that 3/4 patients who presented with a suppressed viral load also had a previous history of AIDS-defining illnesses.

None of our patients showed spontaneous resolution of their RVF following faecal diversion and normalisation of viral control, suggesting that surgical repair of the fistula is required. Several approaches for repair of H-type RVF have been described, including abdominoperineal repair, vestibulo-anal pull-through, limited or formal posterior sagittal anorectoplasty (PSARP), anterior perineal anorectoplasty, and a transanal approach [23] [25–27]. Although no approach has been demonstrated as superior in the literature, our experience supports the use of a transanal technique that preserves the perineal body while mobilising the anterior rectal wall to cover the area of the fistula. By preserving the skin, this approach mitigates the risk of wound sepsis in immunocompromised patients and can yield favourable cosmetic outcomes. While this technique necessitates a partial sacrifice of the dentate line, HIV-associated RVF have been consistently noted to occur at the level of the dentate line, which is therefore already compromised in its integrity and function [5, 11, 15]. In five of our patients, an ischiorectal fat pad was interposed to support the repair with a native, well-vascularised tissue buttress [24]. Other techniques to strengthen repair of acquired or recurrent RVF have been described in the literature, including use of a native bulbocavernosus graft and interposition of a gracilis muscle flap [28–30]. While we did not observe a significant correlation between fat pad interposition and recurrence rates, comparative multi-centre studies with larger sample sizes are required to elucidate the impact of interposition grafts on the risk of complications and long-term outcomes in patients with H-type RVF. In a subset of patients, transanal repair is not possible due to associated perineal disruption secondary to sepsis. These patients may require a posterior sagittal approach with perineal body reconstruction and are likely to have worse outcomes in terms of bowel control due to more extensive damage to the anal sphincters and dentate line.

The most important finding in our study is that good outcomes from surgical repair of acquired RVF in HIV-positive patients can be achieved if viral control is optimised pre-operatively and maintained throughout post-operative follow-up. Our experience represents a departure from studies advocating a conservative approach in the pre-antiretroviral treatment literature and is consistent with the reports of successful repair of HIV-associated RVF after viral suppression in the post-antiretroviral literature [5, 11, 15, 16]. We did not observe a correlation between HIV status at presentation and surgical trajectories. Although most patients presented with stage 3 disease (AIDS), good long-term outcomes without recurrence or complications were observed in patients who achieved viral suppression prior to the point of fistula repair and maintained it thereafter. While we still observed a relatively high recurrence rate of 44% (4/9 patients), recurrence as well as complications such as stenosis or sepsis were mainly observed among patients who did not maintain viral suppression following repair. Due to our small sample size, we were unable to draw a correlation between the timing of recurrences or complications and that of post-operative failures in viral control. With advances in the availability of antiretroviral therapy, it is hoped that HIV-associated RVFs will become a thing of the past and that further studies to investigate the pathophysiology of fistula recurrences in patients with relapses of HIV infection will no longer be required.

## Conclusion

While HIV-associated rectovaginal fistulae remain a challenging condition to manage for paediatric surgeons operating in countries with a high prevalence of HIV infection in children, successful treatment is possible with the combination of antiretroviral treatment and surgery. In our experience, optimisation of HIV control prior to repair of the fistula and maintenance of viral suppression post-operatively are necessary conditions to reduce the risk of recurrence and complications and to achieve good long-term functional outcomes. Larger studies are required to develop consensus on the most appropriate surgical approach to the management of acquired rectovaginal fistulae in children.

## Data Availability

All data supporting the findings of this study are available within the paper and its Appendix.

## Appendix 1 – Classification of HIV disease in the paediatric population

See Tables

**Table 5:** Stage of HIV disease based on age-specific CD4 count (in cells/μl)

Stage	Aged < 1 year	Aged 1–5 years	Aged > 6 years
1 (low risk)	≥ 1500	≥ 1000	≥ 500
2 (moderate)	750–1499	500–999	200–499
3 (AIDS)^a^	< 750	< 500	< 200

^a^If an AIDS-defining condition is identified, the stage is 3 regardless of the CD4 count

5 and

**Table 6:** Acquired immunodeficiency syndrome (AIDS)-defining conditions

Bacterial infections, multiple or recurrent, in children < 6 years
Candidiasis of oesophagus, bronchi, trachea, lungs
Cervical cancer, invasive
Coccidioidomycosis, disseminated or extrapulmonary
Cryptococcosis, extrapulmonary
Cryptosporidiosis, chronic intestinal (> 1 month duration)
CMV disease (other than liver, spleen, or lymph nodes), onset at age > 1 month
CMV retinitis (with loss of vision)
Encephalopathy attributed to HIV
HSV: chronic ulcers (> 1 month duration) or bronchitis, pneumonitis, or esophagitis (onset at age > 1 month)
Histoplasmosis, disseminated or extrapulmonary
Isosporiasis, chronic intestinal (> 1 month duration)
Kaposi sarcoma
Lymphoma, Burkitt (or equivalent term)
Lymphoma, immunoblastic (or equivalent term)
Lymphoma, primary (of brain)
*Mycobacterium avium* complex or *Mycobacterium kansasii*, disseminated or extrapulmonary
*Mycobacterium *tuberculosis of any site, pulmonary, disseminated, or extrapulmonary
*Mycobacterium*, other species or unidentified species, disseminated or extrapulmonary
Pneumocystis jirovecii (previously known as Pneumocystis carinii) pneumonia
Pneumonia, recurrent^c^
Progressive multifocal leukoencephalopathy
Salmonella septicemia, recurrent
Toxoplasmosis of brain, onset at age > 1 month
Wasting syndrome attributed to HIV

Sources: World Health Organization. Updated recommendations on HIV prevention, infant diagnosis, antiretroviral initiation and monitoring. 2021; Available from: https://www.who.int/publications/i/item/9789240022232

Center for Disease Control and Prevention. CDC Pediatric HIV CD4 Cell Count/Percentage and HIV-Related Diseases Categorization. 2022; Available from: https://clinicalinfo.hiv.gov/en/guidelines/pediatric-arv/appendix-c-cdc-pediatric-hiv-cd4-cell

HIV i-Base. CD4 differences between adults and children. 2023; Available from: https://i-base.info/ttfa/section-1/10-differences-between-adults-and-children/

6

## References

[1] Lucas S, Nelson AM (2015) HIV and the spectrum of human disease. J Pathol 235(2):229–241 25251832 10.1002/path.4449

[2] Weledji EP (2013) Human immunodeficiency virus and the anorectum. Alex J Med 49(2):163–167

[3] Barrett WL, Callahan TD, Orkin BA (1998) Perianal manifestations of human immunodeficiency virus infection: experience with 260 patients. Dis Colon Rectum 41(5):606–611 9593244 10.1007/BF02235268

[4] Borgstein ES, Broadhead RL (1994) Acquired rectovaginal fistula. Arch Dis Child 71(2):165–166 7944544 10.1136/adc.71.2.165 PMC1029955

[5] Hyde GA Jr, Sarbah S (1994) Acquired rectovaginal fistula in human immunodeficiency virus-positive children. Pediatrics 94(6 Pt 1):940–941 7971017

[6] Oliver MJ (1995) Acquired rectovaginal fistula. Arch Dis Child 72(3):275 7741586 10.1136/adc.72.3.275-c PMC1511047

[7] Banieghbal B, Fonseca J (1997) Acquired rectovaginal fistulae in South Africa. Arch Dis Child 77(1):94 9279173 10.1136/adc.77.1.91h PMC1717253

[8] Bankole Sanni R et al (1997) Acquired recto-vaginal fistula in children: is HIV infection a cause? Bull Soc Pathol Exot 90(2):111–112 9289247

[9] Schoeman C, Hallbauer U (2001) Rectovaginal fistulas in HIV - infected children. S Afr Med J Suid-Afrikaanse tydskrif vir geneeskunde 91:91–92 11288403

[10] Huang X et al (2021) Acquired rectourethral and rectovaginal fistulas in children: a systematic review. Front Pediatr 9:657251 34026691 10.3389/fped.2021.657251 PMC8138555

[11] Osifo OD, Egwaikhide EA (2011) Acquired rectovaginal fistula in babies of unknown and asymptomatic retroviral positive mothers. J Pediatr Adolesc Gynecol 24(3):e79-82 21256781 10.1016/j.jpag.2010.08.013

[12] Government of South Africa (2022) People of South Africa.; https://www.gov.za/about-sa/people-south-africa-0#:~:text=The%20estimated%20overall%20HIV%20prevalence,the%20population%20is%20HIV%20positive

[13] Johnson LF et al (2020) Steep declines in pediatric AIDS mortality in South Africa, despite poor progress toward pediatric diagnosis and treatment targets. Pediatr Infect Dis J 39(9):843–848 32433224 10.1097/INF.0000000000002680 PMC7958302

[14] Uba AF et al (2004) Acquired rectal fistula in human immunodeficiency virus-positive children: a causal or casual relationship? Pediatr Surg Int 20(11–12):898–901 15480706 10.1007/s00383-004-1285-5

[15] Wiersma R (2003) HIV-positive African children with rectal fistulae. J Pediatr Surg 38(1):62–64 12592620 10.1053/jpsu.2003.50011

[16] Karpelowsky JS et al (2009) Outcomes of human immunodeficiency virus-infected and -exposed children undergoing surgery–a prospective study. J Pediatr Surg 44(4):681–687 19361626 10.1016/j.jpedsurg.2008.08.036

[17] Poenaru D et al (2010) Caring for children with colorectal disease in the context of limited resources. Semin Pediatr Surg 19(2):118–127 20307848 10.1053/j.sempedsurg.2009.11.017

[18] World Health Organization (2023) *The role of HIV viral suppression in improving individual health and reducing transmission*; https://www.who.int/publications/i/item/9789240055179

[19] World Health Organization (2021) Updated recommendations on HIV prevention, infant diagnosis, antiretroviral initiation and monitoring; https://www.who.int/publications/i/item/9789240022232. 33822559

[20] Center for Disease Control and Prevention (2022) CDC Pediatric HIV CD4 Cell Count/Percentage and HIV-Related Diseases Categorization; https://clinicalinfo.hiv.gov/en/guidelines/pediatric-arv/appendix-c-cdc-pediatric-hiv-cd4-cell.

[21] HIV i-Base (2023) CD4 differences between adults and children; https://i-base.info/ttfa/section-1/10-differences-between-adults-and-children/.

[22] Republic of South Africa National Department of Health (2023) ART Clinical Guidelines for the Management of HIV in Adults, Pregnancy and Breastfeeding, Adolescents, Children, Infants and Neonates; Available from: https://knowledgehub.health.gov.za/elibrary/2023-art-clinical-guidelines-management-hiv-adults-pregnancy-and-breastfeeding-adolescents.

[23] Lawal TA et al (2011) Management of H-type rectovestibular and rectovaginal fistulas. J Pediatr Surg 46(6):1226–1230 21683227 10.1016/j.jpedsurg.2011.03.058

[24] Levitt MA et al (2014) The Gonzalez hernia revisited: use of the ischiorectal fat pad to aid in the repair of rectovaginal and rectourethral fistulae. J Pediatr Surg 49(8):1308–1310 25092096 10.1016/j.jpedsurg.2013.10.020

[25] White DW et al (1997) Isolated H-type recto-vaginal fistula associated with a vulval abscess. Pediatr Radiol 27(7):586–587 9211952 10.1007/s002470050189

[26] Tsugawa C et al (1999) Surgical repair of rectovestibular fistula with normal anus. J Pediatr Surg 34(11):1703–1705 10591575 10.1016/s0022-3468(99)90649-8

[27] Meyer T, Höcht B (2009) Congenital H-type anorectal fistula: two case reports. Klin Padiatr 221(1):38–40 17710739 10.1055/s-2007-984373

[28] Zmora O et al (2006) Gracilis muscle transposition for fistulas between the rectum and urethra or vagina. Dis Colon Rectum 49(9):1316–1321 16752191 10.1007/s10350-006-0585-3

[29] Piper HG, Trussler A, Schindel D (2011) Gracilis transposition flap for repair of an acquired rectovaginal fistula in a pediatric patient. J Pediatr Surg 46(8):e37-41 21843707 10.1016/j.jpedsurg.2011.05.004

[30] Cui L et al (2009) Interposition of vital bulbocavernosus graft in the treatment of both simple and recurrent rectovaginal fistulas. Int J Colorectal Dis 24(11):1255–1259 19421760 10.1007/s00384-009-0720-4

